# Topical Delivery of Niacinamide: Influence of Binary and Ternary Solvent Systems

**DOI:** 10.3390/pharmaceutics11120668

**Published:** 2019-12-10

**Authors:** Yanling Zhang, Chin-Ping Kung, Bruno C. Sil, Majella E. Lane, Jonathan Hadgraft, Michael Heinrich, Balint Sinko

**Affiliations:** 1 Department of Pharmaceutics, UCL School of Pharmacy, 29-39 Brunswick Square, London WC1N 1AX, UK; c.kung@ucl.ac.uk (C.-P.K.); majella.lane@btinternet.com (M.E.L.); jonathan.hadgraft@btinternet.com (J.H.); m.heinrich@ucl.ac.uk (M.H.); 2 School of Human Sciences, London Metropolitan University, 166-220 Holloway Road, London N7 8DB, UK; b.dasilvasildossantos@londonmet.ac.uk; 3 Pion Inc., 10 Cook Street, Billerica, MA 01821, USA; balint.sinko@googlemail.com

**Keywords:** in vitro, permeation, niacinamide, solvent, PAMPA, skin

## Abstract

Niacinamide (NIA) is the amide form of vitamin B3 and has been widely used in pharmaceutical and personal care formulations. Previously, we reported a comparative study of NIA permeation from neat solvents using the Skin Parallel Artificial Membrane Permeability Assay (PAMPA) and mammalian skin. A good correlation between NIA permeation in the different models was found. In the present work, ten binary and ternary systems were evaluated for their ability to promote NIA delivery in the Skin PAMPA model, porcine skin and human epidermis. Penetration enhancement was evident for binary systems composed of propylene glycol and fatty acids in human skin studies. However, propylene glycol and oleic acid did not promote enhancement of NIA compared with other systems in the Skin PAMPA model. A good correlation was obtained for permeation data from Skin PAMPA and porcine skin. However, data from the Skin PAMPA model and from human skin could only be correlated when the PG-fatty acid systems were excluded. These findings add to our knowledge of the potential applications of Skin PAMPA for screening dermal/transdermal preparations.

## 1. Introduction

**Figure 1:**
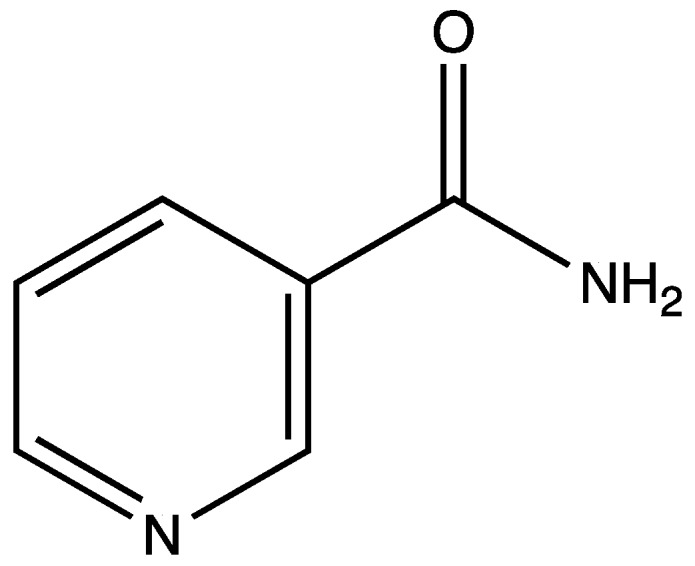
The 2D structure of niacinamide.

Niacinamide (NIA) is the water-soluble form of vitamin B3 (Figure 1). Since its discovery as a “pellagra-preventing” agent, this molecule has been widely used in personal care and cosmetic products [1,2]. NIA has been demonstrated to have a number of anti-inflammatory and photo-protective effects following topical application. The therapeutic benefits of NIA in the management of acne and atopic dermatitis and promotion of the up-regulation of epidermal lipid synthesis have also been reported [3,4]. Mohammed, et al. [5] investigated the influence of NIA on the molecular properties of the human stratum corneum (SC) in vivo. The authors assessed the corneocyte maturity and surface area from NIA treated and untreated (control) areas, as well as trans-epidermal water loss (TEWL). The thickness of the SC before and after the application of NIA was determined using confocal Raman spectroscopy (CRS). The NIA treatment decreased TEWL values, increased the thickness of the SC, and larger and more mature corneocytes were found in the treated versus control sites. More recently, evidence from a phase 3 randomized clinical trial points to NIA having a role in the prevention of non-melanoma skin cancer [6,7].

The discovery and development of robust and accessible human skin surrogates continues to be an area of considerable interest to industry and regulatory authorities. Recently, we reported the use of the Skin Parallel Arterial Membrane Permeability Assay (PAMPA) model to assess in vitro permeation of NIA from a number of solvents, namely, propylene glycol (PG), dimethyl isosorbide (DMI), Transcutol^®^ P (TC), *t*-butyl alcohol (T-BA), PEG 400, PEG 600, and a commercial product containing NIA [8]. In vitro permeation studies were also performed using porcine skin and heat-separated human epidermis. The permeation studies were conducted under finite dose conditions, namely, 5 μL/cm^2^ for human/pig skin and 3 μL/cm^2^ for the Skin PAMPA model. When the permeation data for the models were compared, a correlation coefficient (*R*^2^) of 0.71 for human skin and PAMPA was obtained and the corresponding correlation coefficient for porcine skin and PAMPA was 0.88.

A number of studies have reported that combinations of vehicles and/or chemical penetration enhancers may improve skin uptake and the permeation of actives in a synergistic manner [9,10]. PG is one of the most commonly used glycols in skin preparations [11]. The synergistic enhancement of drug delivery to the skin was reported when PG was employed as a co-solvent in delivery systems [12]. In our previous work, a range of neat solvents were evaluated for NIA skin delivery; both PG and TC were identified as the most promising vehicles for this active [8]. The primary aim of the present work was to examine the efficacy of combinations of PG or TC with other solvents on the skin uptake of NIA. The solvents selected were T-BA, DMI, oleic acid (OA), linolenic acid (LA) and caprylic/capric triglyceride (CCT). These solvents were selected as they encompass varying physicochemical properties and, where known, different mechanisms of skin penetration enhancement [11,13]. A secondary aim was to further explore the feasibility of the Skin PAMPA for screening multi-component formulations.

## 2. Materials and Methods

### 2.1. Materials

NIA, PG, T-BA, OA, LA, high-performance liquid chromatography (HPLC) grade water and methanol, were purchased from Sigma-Aldrich, Dorset, UK. DMI and IPM were supplied from Croda Ltd., Goole, UK. TC and CCT were kind donations from Gattefossé, St. Priest, France. Phosphate buffered saline (PBS) (pH 7.3 ± 0.2 at 25 °C) tablets was supplied by Oxoid, Cheshire, UK. Pre-coated PAMPA Plates (Pion Inc. PN120657), hydration solution, stirring disks and a Gut-Box™ device were obtained from pION Inc. Billerica, MA, USA.

### 2.2. Miscibility and Stability Studies

Miscibility studies of all vehicles were conducted to identify appropriate combinations of solvents for binary and ternary systems. The stability of NIA at a concentration of 5% (*w*/*v*) in PG:T-BA (90:10), PG:OA (10:90), PG:TC (50:50), PG:DMI (50:50), PG:LA (50:50), PG:TC:DMI (50:25:25), TC: T-BA (90:10) and TC:DMI (50:50), TC:CCT:DMI (50:25:25) and TC:PG:DMI (50:25:25) was examined at 32 ± 1 °C up to 72 h [14]. The concentration of NIA was determined at 0, 24, 48 and 72 h. Samples were analyzed using the HPLC method validated earlier with a Kinetix^®^ 5 mm Phenyl-Hexyl 250 × 4.6 mm reversed column (Phenomenex, Macclesfield, UK) with water:methanol (80:20) as the mobile phase. The injection volume was set at 10 μL, and the flow rate of the mobile phase was set to 1 mL/min. The column temperature was set to 30 °C and the UV detection wavelength was 263 nm [8].

### 2.3. The Skin PAMPA Permeation Studies

The influence of binary and ternary solvent systems on the permeation of NIA was initially screened using the Skin PAMPA model. The Skin PAMPA model was hydrated and prepared following the procedure described by Luo, et al. [15]. The permeation studies were conducted using a modified protocol described previously [8]. A dose of 1 μL (corresponding to 3 μL/cm^2^) was used, as it was shown to represent a finite dose of NIA formulations in previous Skin PAMPA studies. To maintain the temperature of the membrane at 32 ± 1 °C, the Skin PAMPA apparatus was incubated in a “Gut-Box™” chamber during the course of permeation studies. Freshly prepared and degassed phosphate buffered saline (PBS, pH 7.3 ± 0.2) solution served as the receptor medium. Samples were collected at 0.1, 0.2, 0.3, 0.7 1, 1.5, 2 and 2.5 h. The concentration of NIA in the receptor medium at each interval was quantified using the HPLC method validated previously [8].

### 2.4. Permeation Studies in Porcine and Human Skin

Vertical glass Franz diffusion cells were used to conduct in vitro permeation studies of NIA in full thickness porcine and heat-separated human skin, as reported previously [16,17]. All permeation studies were conducted under finite dose conditions (5 μL/cm^2^). Porcine tissue was obtained from a local abattoir. The human (female, Caucasian) abdominal tissue from three donors following plastic surgery was obtained from a tissue bank with institutional approval (Research Ethics Committee reference 07/H1306/98). Porcine and human tissue membranes with a diffusion area of around 1 cm^2^ were prepared following the procedures described earlier, and the membrane integrity was examined by a measurement of electrical resistance [8]. The Franz cells were placed into a thermostatically controlled water bath (JB Nova, Grant, Cambridge, UK) set at 36 ± 1 °C during the course of the permeation studies. The NIA formulations were applied to the skin when the temperature of the skin surface had equilibrated to 32 ± 1 °C (TM-22 Digitron digital thermometer, RS Components, Corby, UK). A total of 200 μL of receptor medium was withdrawn and replaced with an equal volume of PBS solution at each sampling interval: 0, 1, 2, 4, 6, 8, 10, 12 and 24 h. At the end of the permeation studies, the receptor phase was removed, and mass balance evaluation was performed following a procedure validated previously [18]. The mass balance method is described in the Supplementary Materials.

### 2.5. Data Analysis

Data were recorded using Microsoft^®^ Excel and analyzed using SPSS^®^ Statistics version 24 (IBM, Feltham, UK) using the procedure described previously [8]. One-way ANOVA with a post hoc Tukey Test was performed for data that met the assumption of normality and homogeneity of variance. The Kruskal–Wallis H Test was used for non-parametric data. A *p*-value of <0.05 was considered as a statistically significant difference. The correlation between the cumulative amounts of NIA that permeated in the Skin PAMPA model and mammalian skin was calculated using the Pearson Product–moment correlation coefficient (*R*^2^) in Microsoft^®^ Excel.

## 3. Results and Discussion

### 3.1. Stability Determination

The stability of NIA in the seven binary and three ternary systems was confirmed. After 72 h, the recovery values of NIA in all tested systems were greater than 91.0%. The stability of NIA in the receptor medium, PBS solution, was confirmed in our earlier work [8]. Stability results are reported in the Supplementary Materials (Figure S1).

### 3.2. Skin PAMPA Permeation Studies

**Figure 2:**
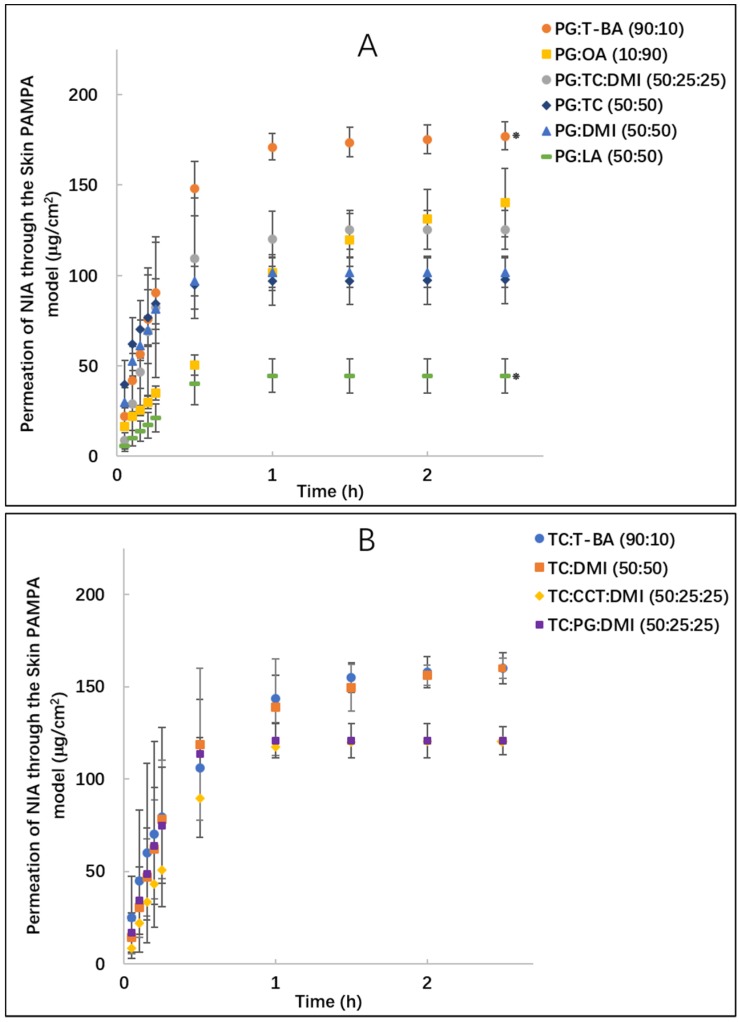
Cumulative permeation of niacinamide (NIA) from binary and ternary solvent systems composed of propylene glycol (PG): *t*-butyl alcohol (T-BA) (●), PG: oleic acid (OA) (■), PG: Transcutol^®^ P (TC) (◆), PG: dimethyl isosorbide (DMI) (▲), PG: linolenic acid (LA) (▄), PG:TC:DMI (50:25:25, ●) (**A**), TC:T-BA(●), TC:DMI(■), TC:PG:DMI (50:25:25, ■) and TC: caprylic/capric triglyceride (CCT):DMI (50:25:25, ◆) (**B**) in the Skin PAMPA model following the application of 1 μL per well (corresponding to 3 μL/cm^2^) of formulations. Each data point represents the mean ± SD, *n* = 4. * *p* < 0.05.

Figure 2A shows the permeation profiles of NIA for PG:T-BA (90:10), PG:OA (10:90), PG:TC (50:50), PG:DMI (50:50), PG:LA (50:50) and PG:TC:DMI (50:25:25) in the Skin PAMPA model. At 2.5 h, the cumulative amounts of NIA that permeated through the membrane ranged from 44.2 to 177.4 μg/cm^2^. PG:T-BA was the most efficient system for NIA delivery compared with all other PG binary or ternary systems (*p* < 0.05). The cumulative amount of NIA delivered through the Skin PAMPA model from neat PG was determined as 163.1 μg/cm^2^ [8]. No difference was evident when comparing NIA permeation from PG:T-BA (90:10) and neat PG (*p* > 0.05). The amount of NIA delivered by PG:OA (10:90), 140.2 ± 18.9 μg/cm^2^, was significantly greater than corresponding values for PG:TC (50:50), PG:DMI (50:50) and PG:LA (50:50) (*p* < 0.05). The ternary system composed of PG, TC and DMI did not enhance the permeation of NIA in Skin PAMPA compared with PG:DMI (50:50) and PG:TC (50:50) (*p* > 0.05). A significantly lower permeation of NIA was observed for PG:LA (50:50), 44.2 ± 9.4 μg/cm^2^, compared with all other binary and ternary systems and neat PG (*p* < 0.05). Formulations containing TC and other solvents were subsequently assessed in the Skin PAMPA model under finite dose conditions (Figure 2B). At 2.5 h, the cumulative amounts of NIA that permeated from TC:T-BA (90:10) and TC:DMI (50:50) were 160.0 ± 8.5 and 159.9 ± 5.4 μg/cm^2^, respectively (*p* > 0.05). The amounts of NIA that permeated from TC:T-BA (90:10) and TC:DMI (50:50) were significantly higher compared with corresponding values for TC:CCT:DMI (50:25:25) and TC:PG:DMI (50:25:25) (*p* < 0.05).

Table 1 summarizes the percentages of NIA permeation from the binary and ternary systems. At 2.5 h, for PG:OA (10:90), PG:T-BA (90:10), PG:TC:DMI (50:25:25), TC:DMI (50:50) and TC:T-BA (90:10), more than 80% of the applied active was delivered into the receptor phase (*p* > 0.05). In the previous study, the cumulative amounts of NIA that permeated for neat TC and PG in the Skin PAMPA model were 184.7 ± 8.9 and 163.1 ± 1.1 μg/cm^2^, accounting for 97% and 95% of the applied amounts, respectively [8]. Compared with neat TC, the TC binary systems did not enhance the permeation of NIA in the Skin PAMPA model (*p* > 0.05). Interestingly, compared with neat PG, the permeation of NIA was enhanced for PG:T-BA (90:10) (*p* < 0.05). However, the permeation of NIA from PG:LA (50:50) was significantly lower than NIA permeation from neat PG (*p* < 0.05).

### 3.3. Porcine Skin Permeation and Mass Balance Studies

**Figure 3:**
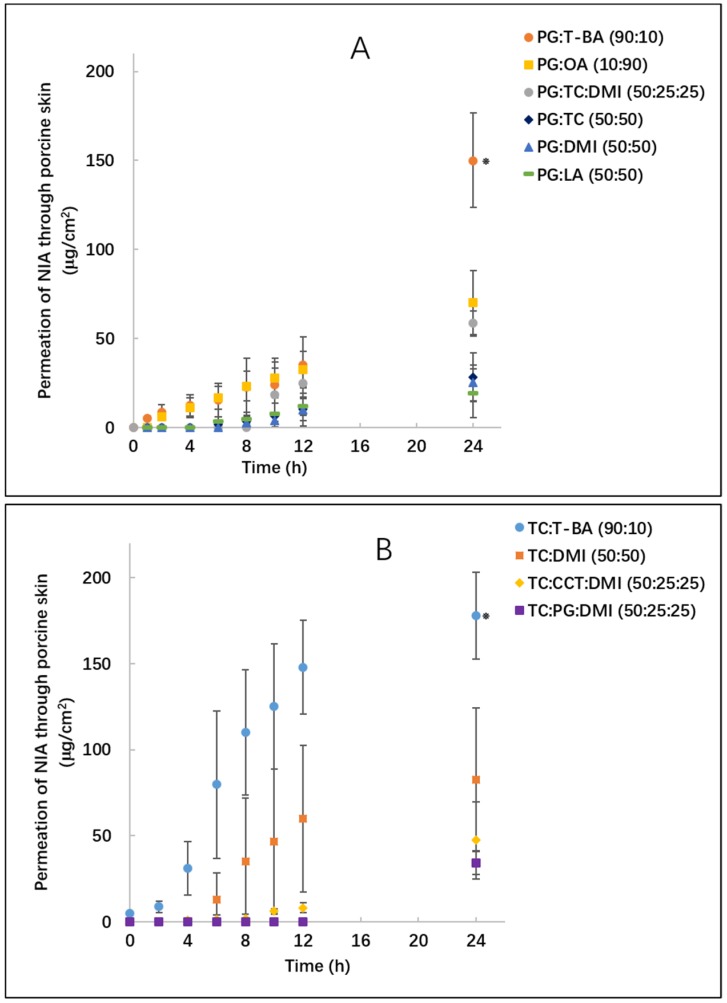
Cumulative permeation of NIA from binary and ternary solvent systems in porcine skin following the application of 5 μL/cm^2^ of formulations. (**A**) shows the permeation profiles of PG:T-BA(●), PG:OA(■), PG:TC(◆), PG:DMI(▲), PG:LA(▄), PG:TC:DMI (50:25:25, ●); (**B**) shows the permeation profiles of TC:T-BA(●), TC:DMI(■), TC:PG:DMI (50:25:25, ■) and TC:CCT:DMI (50:25:25, ◆). Each data point represents the mean ± SD, *n* = 4. * *p* < 0.05.

The various binary and ternary solvent systems were examined for their influence on NIA permeation using porcine skin under finite dose conditions (5 μL/cm^2^). Figure 3A,B show the permeation profiles for the binary formulations composed of PG or TC. For PG:T-BA (90:10), NIA permeation was detected after 1 h and this system delivered a significantly higher amount of NIA through the tissue compared with other formulations, namely, 150.3 ± 26.5 μg/cm^2^ at 24 h (*p* < 0.05). The cumulative amounts of NIA that permeated from PG:OA (10:90) and PG:TC:DMI (50:25:25) were 70.2 ± 18.1 and 58.4 ± 7.0 μg/cm^2^, respectively (*p* > 0.05). The cumulative permeation of NIA from PG: LA (50:50) was 19.3 ± 13.7 μg/cm^2^, a value that was significantly lower than PG:T-BA (90:10). The amounts of NIA that permeated through porcine skin from binary and ternary formulations of TC ranged from 34.1 to 178.0 μg/cm^2^ (Figure 3B). The cumulative amounts of NIA that permeated from neat TC and PG were previously reported as 95.1 and 46.0 μg/cm^2^ in pig skin, respectively [8]. At 24 h, a higher cumulative permeation of NIA was evident for TC:T-BA (90:10) compared with neat TC or other assessed binary and ternary systems containing TC (*p* < 0.05).

The overall recoveries in the mass balance studies for PG:OA (10:90), PG:DMI (50:50), PG:TC (50:50), TC:DMI (50:50) and PG:TC:DMI were <85% (Table 2). These results are consistent with the findings of Haque et al. [18]. As the stability of NIA in all formulations was confirmed, it is possible that the lower recovery might be attributed to the chemical derivatization of this molecule during the permeation process [19]. The higher total recovery in binary TC-T-BA systems may reflect the potential of TC:T-BA (90:10) to prevent the chemical derivatization of NIA. However, this hypothesis needs to be probed further. For PG:T-BA (90:10) and TC:T-BA (90:10), lower percentages of NIA were recovered from the skin surface compared with all other binary and ternary systems, accounting for 3.5% and 6.1% of applied NIA amounts, respectively (*p* < 0.05). After 24 h, for all formulations, skin extraction values ranged from 3.2% to 32.9% of the applied amounts. PG:TC (50:50) significantly increased the percentage of NIA deposited in porcine skin compared with neat PG (*p* < 0.05), while no difference was detected when compared with TC (*p* > 0.05). The skin retention values observed for the three tested ternary systems were not significantly different (*p* > 0.05). Interestingly, comparing the distribution of NIA in the tissue for binary and ternary systems, a higher skin retention was evident for TC:CCT:DMI (50:25:25) than for TC:DMI (50:50) (*p* < 0.05). CCT is a triglyceride comprising a mixture of caprylic and capric acid esters and has been widely used in cosmetic products as an emollient [20]. Leopold and Lippold [21] investigated the mechanism of the penetration enhancing effects of several lipophilic vehicles including CCT using Differential Scanning Calorimetry (DSC). These authors suggested that any penetration enhancement effects of CCT are probably caused by the dissolution or extraction of the stratum corneum lipids. The systems composed of CCT examined here appear promising for the dermal delivery of NIA, and these formulations were further evaluated using human skin.

**Figure 4:**
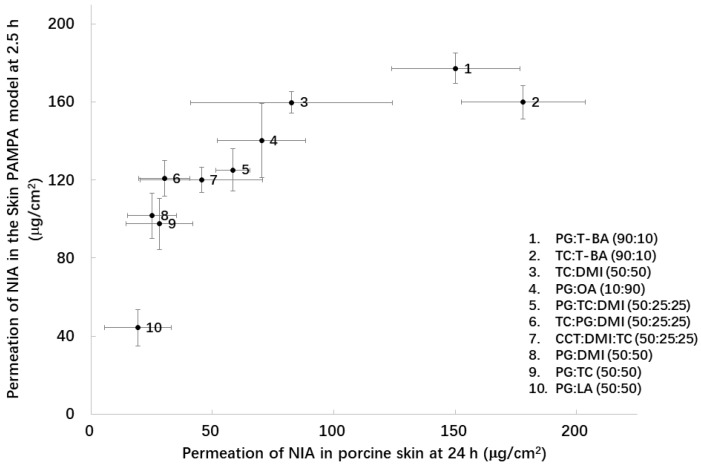
Cumulative amounts of NIA that permeated from assessed binary and ternary solvent systems in the Skin PAMPA model at 2.5 h plotted against the corresponding values observed in porcine skin at 24 h. Each data point represents the mean ± SD, *n* = 4.

As shown in Figure 4, 19.3–178.0 μg/cm^2^ of NIA penetrated through porcine skin at 24 h, while 44.2–177.4 μg/cm^2^ was delivered in the Skin PAMPA model at 2.5 h. A correlation coefficient (*R*^2^) of 0.63 was determined for the linear regression of the data (Figure 4). The value was lower compared with the correlation coefficient determined between the permeation data in Skin PAMPA and porcine skin for the single solvents under finite dose conditions, *R*^2^ = 0.88 [8]. This may reflect the different interactions of the more complex vehicles studied here in the Skin PAMPA lipids compared with the single solvents studied previously.

### 3.4. Human Skin Permeation and Mass Balance Studies

**Figure 5:**
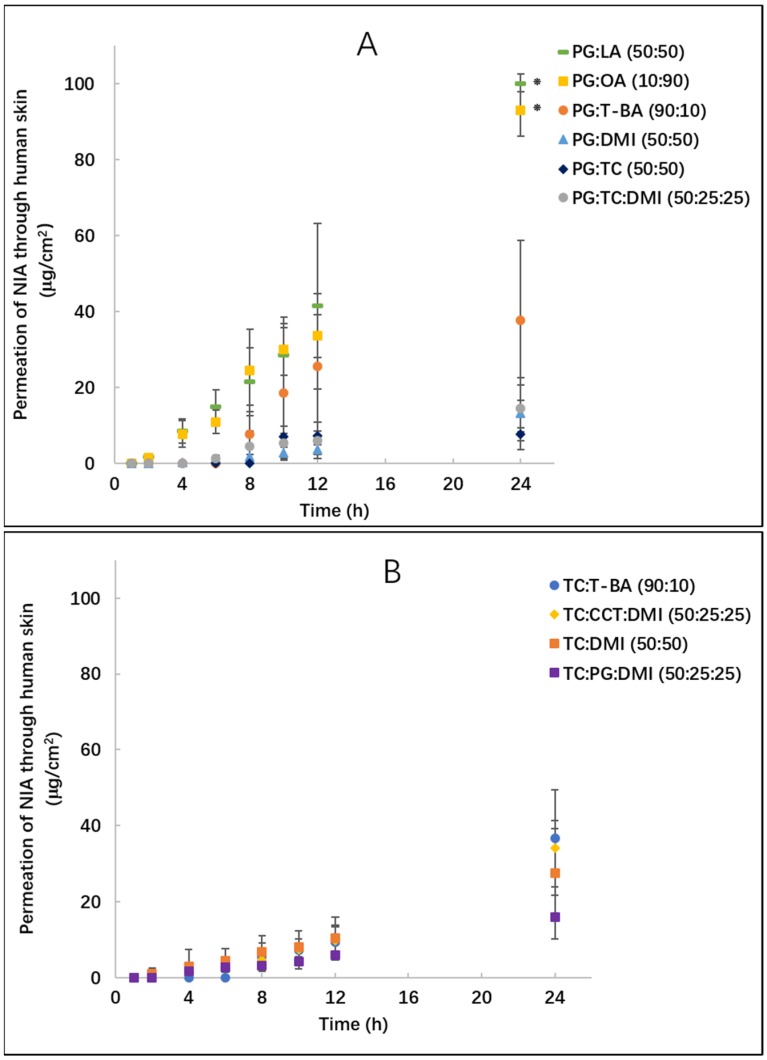
Cumulative permeation of NIA from binary and ternary solvent systems in human skin following the application of 5 μL/cm^2^ of formulation. (**A**) shows the permeation profiles of PG:T-BA(●), PG:OA(■), PG:TC(◆), PG:DMI(▲), PG:LA(▄), PG:TC:DMI (50:25:25, ●); (**B**) shows the permeation profiles of TC:T-BA(●), TC:DMI(■) TC:PG:DMI (50:25:25, ■) and TC:CCT:DMI (50:25:25, ◆). Each data point represents the mean ± SD, *n* = 4. * *p* < 0.05.

NIA permeation was further investigated using heat-separated human epidermis. The permeation profiles of NIA for these experiments are shown in Figure 5. For the two binary systems, PG:LA (50:50) and PG:OA (10:90), permeation of NIA was detected 2 h after application. At 24 h, significantly higher amounts of NIA were delivered from PG:LA (50:50) and PG:OA (10:90) compared with all other binary and ternary systems (*p* < 0.05), with cumulative permeation values of 100.4 ± 2.4 and 93.3 ±2.4 μg/cm^2^, respectively. The cumulative amounts of NIA that permeated in human skin for neat T-BA, DMI, PG or TC were reported previously as 50.8, 15.0, 1.8 and 16.4 μg/cm^2^ [8]. A more efficient skin penetration enhancement of NIA is clearly evident for PG:LA and PG:OA compared with these neat solvents (*p* < 0.05). No significant increase was observed for NIA permeation from TC binary and ternary systems compared with neat TC (*p* > 0.05).

Table 3 summarizes the mass balance results of NIA for the human skin studies. The amounts of NIA remaining on the skin surface range from 27% to 75% of the amounts applied, respectively. No difference was detected comparing the percentage of NIA recovered from the skin surface for the different formulations (*p* > 0.05). The skin retention of NIA for PG:LA (50:50) was determined as 3.4% of the applied dose. This value was significantly lower (*p* < 0.05) compared with the skin extraction values for TC:T-BA (34.7%) and TC:CCT:DMI (29.7%). The percentage of NIA extracted from human epidermis for neat TC was 32.9% [8]. The corresponding value for TC:T-BA (90:10) was comparable to the value for neat TC (*p* > 0.05). As for the percentage permeation, higher values were observed for PG:LA (50:50) (46.1%) and PG:OA (10:90) (40.7%) compared to all other binary and ternary systems (*p* < 0.05). There was no significant increase in the percentage permeation of NIA from TC binary systems compared with neat TC (*p* > 0.05). Statistical analysis confirmed the penetration enhancement of NIA for the PG/fatty acids (LA and OA) compared with neat PG [8] (*p* < 0.05). However, in porcine skin studies, the percentage permeation of NIA for PG:LA (50:50) was significantly lower than for neat PG and PG:OA (10:90) (*p* < 0.05).

Fatty acids have been reported to be skin permeation enhancers for a range of substances possessing varying physicochemical properties [11,22,23]. Recently, Pham, et al. [24] applied ^13^C polarization transfer solid-state nuclear magnetic resonance (NMR) to investigate the effects of OA on SC molecular components. The authors mixed 30 mg of dry SC powder with 5% of OA and then hydrated the mixture with water. The samples were incubated at 32 °C for 24 h before NMR measurement. It was suggested that OA promoted increased mobility of both stratum corneum protein components and the cholesterol chain segments in the intercellular lipid domains. However, the exact synergistic mechanism of PG and fatty acids remains unclear. It may also be hypothesized that the fatty acid first penetrates into the stratum corneum and contributes to increased lipid fluidity, which allows the diffusion of PG with dissolved NIA.

In the present study, there was no significant difference between the permeation in human skin and porcine skin for NIA in TC:DMI (50:50) PG:DMI (50:50), TC:CCT:DMI (50:25:25) (*p* > 0.05). A higher permeation of NIA in porcine skin compared to human skin was evident for PG:T-BA (90:10), PG:DMI (50:50), PG:TC (50:50), PG:TC:DMI (50:25:25), TC:T-BA (90:10) and TC:PG:DMI (50:25:25) (*p* < 0.05). However, a lower permeation of NIA in porcine skin compared to human tissue was evident for PG:LA (50:50) and PG:OA (10:90) (*p* < 0.05). Similar results have been reported by other researchers for other actives. Shin, et al. [25] noted higher flux values of fentanyl for human skin compared with porcine skin. Although pig skin has generally been considered to be more permeable than human skin [26,27,28], the permeability may be dependent on the molecule of interest. Jung and Maibach [29] reviewed 46 studies which evaluated the permeation of 77 chemicals using both porcine and human skin; for 16 chemicals, permeation was greater in human skin permeation compared with porcine skin.

**Figure 6:**
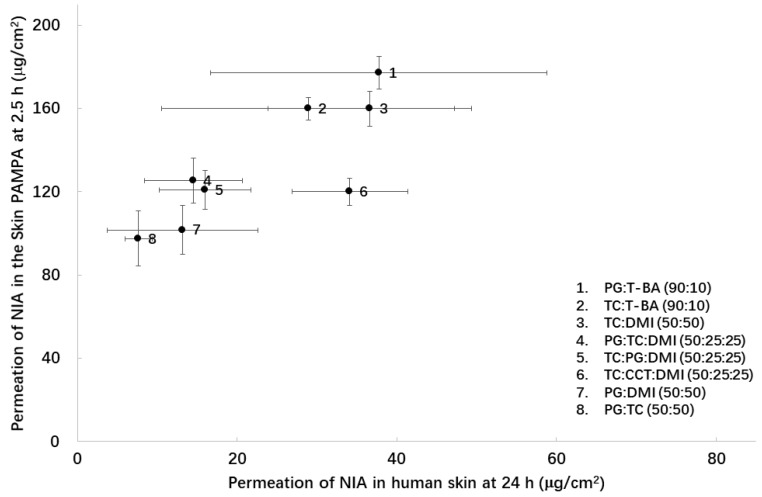
Cumulative permeation data of NIA from assessed binary and ternary solvent systems in the Skin PAMPA model at 2.5 h, plotted against the corresponding values observed in human skin at 24 h. Each data point represents the mean ± SD, *n* = 4.

The cumulative permeation of NIA in human skin ranged from 14.5 to 100.4 μg/cm^2^; the corresponding values determined in the Skin PAMPA model ranged from 44.2 to 177.4 μg/cm^2^. Higher NIA permeation was observed in the artificial membrane compared with human epidermis for PG:T-BA (90:10), TC:T-BA (90:10), TC:DMI (50:50), PG:OA (10:90), PG:TC:DMI (50:25:25), TC:CCT:DMI (50:25:25), PG:DMI (50:50) and PG:TC (50:50) (*p* < 0.05). Attempts to fit all the permeation data from the Skin PAMPA model and human epidermis were not successful. However, excluding results for PG:LA (50:50) and PG:OA (10:90), an *R*^2^ value of 0.68 was obtained for the linear regression of the data (Figure 6). The higher permeability of the Skin PAMPA studies observed in this study is consistent with findings reported by others. Luo et al. [15] investigated the permeation of ibuprofen from PG, PEG 300, commercial gel and spray formulations in the Skin PAMPA model, porcine and human skin. The authors reported the more permeable nature of the Skin PAMPA model compared to human skin. Although the authors did not correlate the permeation data obtained from different models, they reported formulations that delivered a higher amount of ibuprofen in the Skin PAMPA model were also more efficient in human skin. In our previous work, NIA permeation from neat T-BA, DMI, TC, PG, PEG 400 and PEG 600 and one commercial product in the Skin PAMPA model were correlated with porcine/human skin [8]. A correlation coefficient (*R*^2^) of 0.71 was established for the Skin PAMPA with human skin under finite dose conditions. The corresponding correlation between PAMPA model and porcine skin was determined as 0.88. In this work, a correlation of NIA permeation data in human skin and PAMPA model could only be obtained when the permeation data for PG-OA and PG-LA were excluded. Statistical analysis confirmed that the permeation of NIA from PG:LA (50:50) was significantly lower than neat PG (*p* < 0.05) in the Skin PAMPA. Additionally, in the Skin PAMPA model, the permeation of NIA from PG:OA (10:90) was significantly higher compared with PG:LA (50:50) (*p* < 0.05), while no difference was observed between these two systems in human epidermis (*p* > 0.05).

To our knowledge, this is the first study that has investigated the permeation enhancement of fatty acids with PG in the Skin PAMPA model. The results obtained in this study suggested that the Skin PAMPA model is not a suitable model for screening such combinations. The Skin PAMPA model uses a homogeneous filter-impregnated lipid mixture membrane, containing ~50% of synthesized ceramides (Certramide), 25% of stearic acid and 25% of cholesterol [30]. The lower permeation of binary solvent systems composed of PG and fatty acid may reflect the interaction of the lipid components with those formulations. Both OA and LA are unsaturated C_18_ fatty acids [31]. These two fatty acids have a double bond in the conformation [32]. It is possible that OA and LA integrated into the PAMPA membrane and modified the lateral chain packing in the lipid matrix. This may lead to changes in the physicochemical properties and permeability of this artificial model. Further studies are needed to probe this theory.

## 4. Conclusions

Previous work investigated the dermal delivery of NIA from a series of single solvents. In the present work, the influence of binary and ternary solvents on the in vitro permeation of NIA was assessed in the Skin PAMPA model, porcine skin and heat separated human epidermis. The higher skin penetration of NIA was evident in human skin for the binary systems composed of PG with fatty acids (PG-OA, PG-LA) compared with neat solvents investigated previously. A correlation of 0.63 was determined for the permeation data of binary and ternary systems in Skin PAMPA and in porcine skin. Excluding the PG-OA and PG-LA systems, a correlation of 0.68 (*R*^2^) was also evident between the permeation data obtained in the Skin PAMPA model and human epidermis. Further studies to investigate the interactions between the Skin PAMPA model and the fatty acids OA and LA are needed to better understand the potential of this model in the assessment of transdermal/dermal products. Ultimately, a robust, efficient, accessible artificial skin permeability assay would benefit the regulatory authorities, the pharmaceutical industry and the patients.

## Figures and Tables

**Table 1:** Percentage permeation of NIA from the binary systems in the Skin PAMPA model (*n* = 4, mean ± SD).

Formulation	Percentage Permeation (%) at 2.5 h
PG:LA (50:50)	26.9 ± 5.7
PG:DMI (50:50)	66.0 ± 6.9
PG:TC (50:50)	68.0 ± 12.3
PG:OA (10:90)	95.2 ± 13.8
PG:T-BA (90:10)	103.7 ± 0.5
TC:T-BA (90:10)	97.3 ± 5.1
TC:DMI (50:50)	97.2 ± 3.3
TC:PG:DMI (50:25:25)	77.5 ± 8.2
TC:CCT:DMI (50:25:25)	75.3 ± 4.9
TC:CCT:DMI (50:25:25)	81.2 ± 3.2

**Table 2:** The results of the mass balance studies after the permeation studies using porcine skin under finite dose conditions (*n* = 4, mean ± SD).

Formulation	Washing %	Extraction %	Permeation %	Total %
PG:DMI (50:50)	47.1 ± 5.4	22.9 ± 13.2	11.5 ± 3.9	81.5 ± 5.6
PG:OA (10:90)	37.1 ± 5.4	9.6 ± 1.0	30.2 ± 7.2	76.8 ± 5.9
PG:TC (50:50)	43.4 ± 3.9	23.4 ± 5.6	13.4 ± 6.9	80.2 ± 6.7
PG:LA (50:50)	55.6 ± 12.3	20.6 ± 4.4	9.2 ± 6.6	85.4 ± 7.9
PG:T-BA (90:10)	3.5 ± 0.8	10.1 ± 7.6	71.3 ± 12.6	84.9 ± 7.1
TC:T-BA (90:10)	6.1 ± 5.2	15.3 ± 11.9	79.5 ± 12.0	100.9 ± 13.8
TC:DMI (50:50)	35.3 ± 13.6	10.7 ± 3.5	35.2 ± 16.0	81.2 ± 1.8
PG:TC:DMI (50:25:25)	36.1 ± 8.5	18.8 ± 8.8	25.1 ± 3.8	80.1 ± 9.7
TC:CCT:DMI (50:25:25)	39.8 ± 15.1	28.0 ± 12.8	18.2 ± 7.6	86.0 ± 8.9
TC:PG:DMI (50:25:25)	43.2 ± 4.0	32.9 ± 13.8	13.2 ± 1.9	89.2 ± 16.8

**Table 3:** The results of mass balance studies after permeation studies using human skin under finite dose conditions (*n* = 4, mean ± SD).

Formulations	Washing%	Extraction%	Permeation%	Total%
PG:DMI (50:50)	59.6 ± 7.1	17.4 ± 3.9	5.6 ± 4.0	82.6 ± 7.8
PG:OA (10:90)	37.7 ± 6.7	9.2 ± 0.6	40.7 ± 6.5	87.6 ± 0.8
PG:TC (50:50)	69.8 ± 7.8	29.0 ± 18.5	4.4 ± 2.3	103.1 ± 13.4
PG:LA (50:50)	27.1 ± 5.6	3.4 ± 2.0	46.1 ± 5.1	76.5 ± 5.1
PG:T-BA (90:10)	52.8 ± 9.0	20.1 ± 6.0	12.3 ± 4.2	85.2 ± 10.9
TC:T-BA (90:10)	39.8 ± 16.5	34.7 ± 10.5	15.6 ± 5.6	90.1 ± 5.2
TC:DMI (50:50)	75.1 ± 11.7	13.2 ± 4.7	10.2 ± 4.4	98.5 ± 11.7
PG:TC:DMI (50:25:25)	64.6 ± 4.5	12.2 ± 2.0	6.2 ± 2.3	83.0 ± 4.2
TC:CCT:DMI (50:25:25)	44.9 ± 6.0	29.7 ± 4.9	12.9 ± 3.3	87.5 ± 4.9
TC:PG:DMI (50:25:25)	66.5 ± 9.1	14.6 ± 3.4	6.6 ± 2.5	87.8 ± 6.7

## Supplementary Materials

The following are available online at https://www.mdpi.com/1999-4923/11/12/668/s1, Figure S1: Results of stability studies. Percentage of NIA recovered from tested binary and ternary solvent systems at 24, 48 and 72 h at 32 ± 1 °C (*n* = 3, mean ± SD).

## List of Abbreviations

NIA	Niacinamide
PAMPA	Parallel Artificial Membrane Permeability Assay
ANOVA	One-way analysis of variance
PG	Propylene glycol
DMI	Dimethyl isosorbide
OA	Oleic acid
LA	Linolenic acid
T-BA	*t*-butyl alcohol
TC	Transcutol^®^ P
CCT	Caprylic/capric triglyceride
PEG	Polyethylene glycol
CIRP	Cosmetic Ingredient Review Panel
SD	Standard deviation
SC	Stratum corneum
NMR	Nuclear magnetic resonance
FTIR	Fourier transform infrared spectroscopy
