# Supplementary material for: Topical Delivery of Niacinamide: Influence of Binary and Ternary Solvent Systems
Source: Pharmaceutics. 2019 Dec 10;11(12):668. doi: 10.3390/pharmaceutics11120668 (PMC6956018; doi:10.3390/pharmaceutics11120668)
Supplement: Supplementary file 1 [file pharmaceutics-11-00668-s001.pdf]

pharmaceutics

Supplementary Materials: Topical Delivery of Niacinamide: Influence of Binary and Ternary Solvent Systems

Yanling Zhang, Chin-Ping Kung, Bruno C. Sil, Majella E. Lane, Jonathan Hadgraft, Michael Heinrich and Balint Sinko

1. Method of Mass Balance Study

The skin surface was washed using 1 mL of methanol: water (50:50) solution for three-time. The skin surface was swabbed using a cotton bar. The skin was subsequently removed from the Franz cells and cut into small pieces using surgical scissors. The cotton bar and tissue were collected and placed into Eppendorf® tubes with 1 mL washing solution. NIA in all these samples was extracted by placing tubes in an orbital shaker maintained at 32 ± 1 °C overnight. All the samples were centrifuged at 5000 g at 32 °C for 15 min. Aliquots were withdrawn from the supernatant portion. The concentration of NIA was quantified using HPLC. Samples were diluted when necessary. Mass balance results were calculated using Equation (S1), where T represents the total recovery of NIA, W represents the recovered percentage of NIA from the skin surface, E represents the percentage of NIA extracted from the skin membrane and P represents the percentage value of NIA permeated into the receptor medium.

$$T = W + E + P \quad \text{(S1)}$$

2. Results of Stability Study

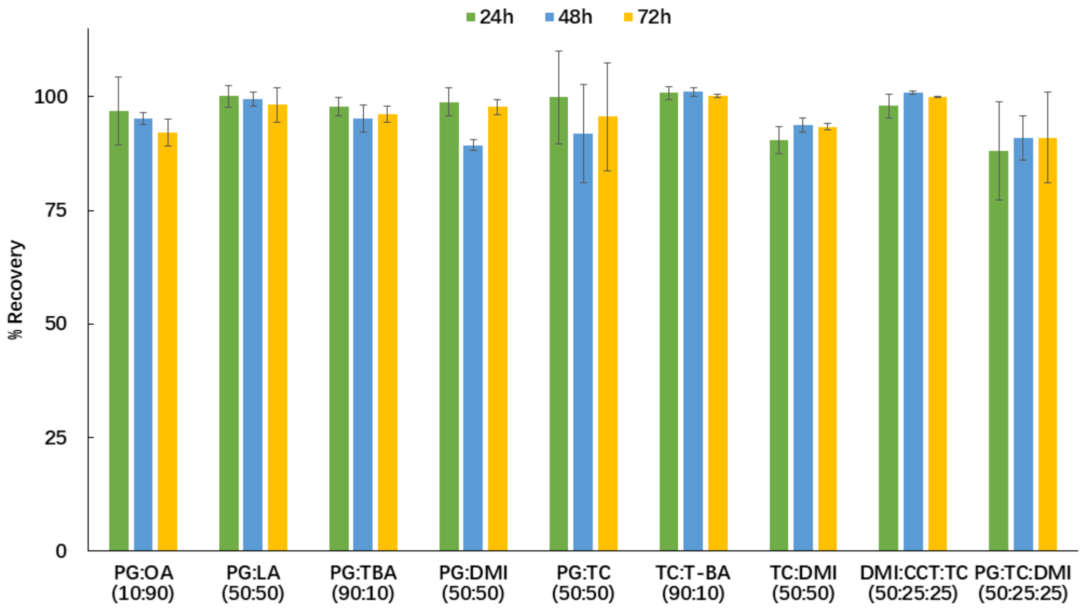

Figure S1. Results of stability studies. Percentage of NIA recovered from tested binary and ternary solvent systems at 24, 48 and 72 h at 32 ± 1 °C (n = 3, mean ± SD).
